# Lifetime risk assessment in cholesterol management among hypertensive patients: observational cross-sectional study based on electronic health record data

**DOI:** 10.1186/s12875-020-01138-5

**Published:** 2020-04-14

**Authors:** Aapo Tahkola, Päivi Korhonen, Hannu Kautiainen, Teemu Niiranen, Pekka Mäntyselkä

**Affiliations:** 1 University of Eastern Finland, Institute of Public Health and Clinical Nutrition, Kuopio, Finland; 2 Health Centre of Jyväskylä Cooperation Area, Jyväskylä, Finland; 3 University of Turku, Turku, Finland; 4 Medcare Oy, Äänekoski, Finland; 5 National Institute for Health and Welfare, Helsinki, Finland; 6 Department of Medicine, Turku University Hospital and University of Turku, Turku, Finland; 7 Primary Health Care Unit, Kuopio University Hospital, Kuopio, Finland

**Keywords:** Dyslipidemia, Hypertension, Lifetime risk, Lipid-lowering medication, LDL-C, Target

## Abstract

**Background:**

In hypertensive patients, reducing plasma low-density lipoprotein cholesterol level (LDL-C) is one of the main interventions for preventing chronic cardiovascular diseases (CVD). However, LDL-C control remains generally insufficient, also in patients with hypertension. We analyzed Electronic Health Record (EHR) data of 7117 hypertensive patients to find the most potential age and sex subgroups in greatest need for improvement in real life dyslipidemia treatment. Taking into account the current discussion on lifetime CVD risk, we focused on the age dependence in LDL-C control.

**Methods:**

In this observational cross-sectional study, based on routine electronic health record (EHR) data, we investigated LDL-C control of hypertensive, non-diabetic patients without renal dysfunction or CVD, aged 30 years or more in Finnish primary care setting.

**Results:**

More than half (54% of women and 53% of men) of untreated patients did not meet the LDL-C target of < 3 mmol/l and one third (35% of women and 33% of men) of patients did not reach the target even with the lipid-lowering medication (LLM). Furthermore, higher age was strongly associated with better LDL-C control (*p* < 0.001) and lower LDL-C level (p < 0.001) in individuals with and without LLM. Higher age was also strongly associated with LLM prescription (p < 0.001). In total, about half of the patients were on LLM (53% of women and 51% of men).

**Conclusions:**

Our findings indicate that dyslipidemia treatment among Finnish primary care hypertensive patients is generally insufficient, particularly in younger age groups who might benefit the most from CVD risk reduction over time. Clinicians should probably rely more on the lifetime risk of CVD, especially when treating working age hypertensive patients.

## Background

Dyslipidemia increases the risk for cardiovascular diseases (CVD) considerably, especially when combined with other risk factors, such as hypertension [1, 2]. The relationship between dyslipidemia and CVD is particularly strong with plasma low-density lipoprotein cholesterol level (LDL-C) as every 1 mmol/L increase in LDL-C is associated with 28% risk increase in coronary heart disease mortality [3]. Reducing LDL-C is therefore one of the central focuses in preventing CVD, also in hypertensive patients.

Treatment decisions should always be based on the total risk for CVD. The majority of hypertensive patients without CVD, diabetes or moderate to severe chronic kidney disease (CKD; stages 3–5) have low to moderate risk for CVD [3]. According to the 2016 European Society of Cardiology (ESC) and the European Atherosclerosis Society (EAS) Guidelines for the Management of Dyslipidaemias, the target for individuals at low or moderate total CVD risk for treatment is LDL-C < 3 mmol/l [3]. Lifestyle changes are essential as a first approach but with hypertensive patients, who have moderate-high CV risk, lipid-lowering therapy is justified [4–9].

Recent studies demonstrate that LDL-C control remains quite insufficient in Finland and other European countries, even among high CVD risk populations [10, 11].. LDL-C control seems to be especially poor in younger age groups in many countries and patient groups [11–14]. This is an important signal, given the accumulating evidence on the benefits of adequate LDL-C –control over time and growing interest on life-long prevention of CVD [15–19].

The aim of this study was to analyze routine EHR data of hypertensive patients in order to find the most potential age and sex subgroups in greatest need for improvement in dyslipidemia treatment. We were particularly interested on potential age dependence in LDL-C control based on the viewpoint of lifetime CVD risk.

## Methods

In this observational cross-sectional study using routinely collected health care data together with laboratory data, we investigated the level of plasma LDL-C among Finnish hypertensive patients. The most recent LDL-C measurement available for each individual patient was used in the analysis. Permission for this study was obtained from the research committee of Health Centre of Jyväskylä Cooperation Area and it complies with the Declaration of Helsinki. Ethics approval was deemed unnecessary according to Finnish legislation [20].

### Setting

The study was conducted in Finnish public primary care setting in Jyväskylä area. The comprehensive electronic health record data of total population of 155,411 was screened on 24th of May, 2016. The data included laboratory and medication data between between January 2011 and December 2015.

### Patients

**Fig. 1:**
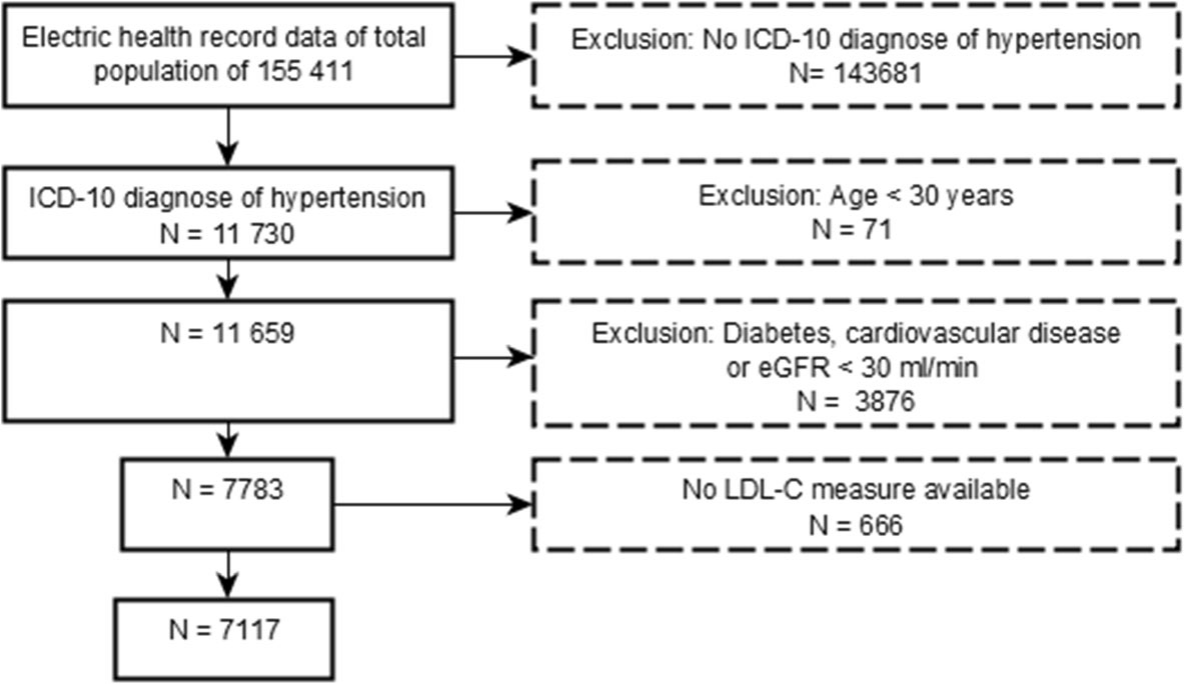
The screening process of the study. eGFR: Estimated glomerulus filtration rate; LDL-C: Low-density lipoprotein

We included all hypertensive patients aged 30 years or more. The identification of patients was based on the personal identity code. Every citizen of Finland has a personal identity code that remains unchanged throughout person’s lifetime. We included patients with presence of ≥1 claims with International Classification of Diseases, Tenth Revision (ICD-10) code for essential hypertension (I10.xx) [21]. We included diagnostic codes starting from the year 2011. Since the diagnostic criteria for hypertension has changed from time to time and the diagnoses of hypertension for individual study patients has been made over a relatively long time period, it is not possible to define specific diagnostic values for hypertension in our study patients. Exclusion criteria were: (1) diabetes (E10.xx-E14.xx), (2) cardiovascular disease (ischemic heart disease I20.xx – I25.xx, stroke I60.xx – I63.xx, peripheral artery disease I70.xx – I79.xx), (3) severe renal dysfunction and (4) age under 30 years. The presence of severe renal dysfunction was based on an estimated glomerulus filtration rate (eGFR < 30 ml/min/1.73 m2) [22]. Patients with diabetes were excluded because our data did not allow us to identify patients with microvascular diabetic complications and, therefore, setting the right target level for diabetics was not possible. Patients < 30 years of age were excluded because the small amount of patients (*n* = 71) made it impossible to carry out a feasible analysis. The screening process is presented in Fig. 1. LDL-C target was considered to be < 3 mmol/l, according to European and Finnish treatment guidelines [3, 23].

### Statistical analyses

The characteristics are presented as means with standard deviation (SD) for continuous variables and as frequencies with percentages for categorical variables, according to gender. Statistical comparisons between groups were done using chi-square test, t-test and generalized linear models (analysis of variance and logistic models). Tests for interactions between LLM-group or gender and age group were conducted by adding a multiplicative term between group or gender and the age group. The normality of the variables was tested by using the Shapiro-Wilk W test. Adjustment for multiple comparisons was considered unnecessary. The Stata 14.1, StataCorp LP (College Station, TX, USA) statistical package was used for the analysis.

## Results

A total of 7117 hypertensive patients were identified from the EHR data. Table 1 displays the patients’ co-morbidities, medication usage and LDL-C levels according to gender.

**Table 1:** Characteristics of study patients

	Women *N* = 4344	Men *N* = 2773	*P*-value
**Demographics**			
Mean age (years), mean (SD)	70 (12)	66 (12)	< 0.001
**Medication (ATC), n (%)**			
Lipid-lowering medication (C10)	2310 (53)	1400 (50)	0.027
Other cardiac therapy e.g. antiarrhythmics, nitrates (C01)	936 (22)	429 (15)	< 0.001
ACE-inhibitors, Angiotensin II antagonists (C09)	3531 (81)	2330 (84)	0.003
Diuretics (C03)	1370 (32)	538 (19)	< 0.001
Beta blockers (C07)	2477 (57)	1312 (47)	< 0.001
Calcium Channel Blockers (C08)	2150 (49)	1379 (50)	0.85
Other antihypertensives (C02)	114 (3)	73 (3)	0.97
**Co-morbidity (ICD-10), n (%)**			
Heart failure (I50)	95 (2)	31 (1)	< 0.001
Atrial fibrillation and flutter (I48)	295 (7)	207 (7)	0.28
**Plasma LDL-C level, mean (SD)**			
All	2.88 (0.88)	2.85 (0.88)	< 0.001^a^
With LLM	2.75 (0.97)	2.68 (0.98)	< 0.001^a^
Without LLM	3.03 (0.73)	3.01 (0.73)	0.25^a^

*Abbreviations*. *LLM* lipid lowering medication, *LDL-C* low-density lipoprotein

^a^Adjusted for age

In total, 65% of hypertensive women and 67% of hypertensive men treated with LLM reached the LDL-C target < 3 mmol/l. Without LLM, the proportion of patients reaching the target was even lower (46% of women and 47% of men). Of all patients, 56% of hypertensive patients reached the LDL-C target. The proportions of individuals reaching treatment target with and without medication is presented in Table 2.

**Table 2:** Proportion of individuals reaching LDL-C target

	Age 30–49	Age 50–59	Age 60–69	Age 70–79	Age 80 -	Total
**LDL-C TARGET REACHED**						
**Women without LLM**						
Yes, n (%)	119 (50.4)	132 (37.5)	250 (41.5)	235 (47.2)	192 (55.7)	928 (45.6)
**Women with LLM**						
Yes, n (%)	15 (55.6)	75 (48.1)	436 (62.1)	595 (70.0)	383 (66.6)	1504 (65.1)
**Men without LLM**						
Yes, n (%)	80 (41.2)	100 (36.8)	207 (46.1)	175 (54.4)	90 (66.2)	652 (47.5)
**Men with LLM**						
Yes, n (%)	27 (42.9)	90 (54.9)	362 (68.2)	337 (71.6)	121 (70.8)	937 (66.9)
**LIPID-LOWERING MEDICATION**						
**Women**						
Yes, n (%)	27 (10.3)	156 (30.7)	702 (53.8)	850 (63.0)	575 (62.5)	2310 (53.2)
** Men**						
Yes, n (%)	63 (24.5)	164 (37.6)	531 (54.2)	471 (59.4)	171 (55.7)	1400 (50.5)

*Abbreviations*. *LDL-C* low-density lipoprotein, *LLM* lipid-lowering medication

**Fig. 2:**
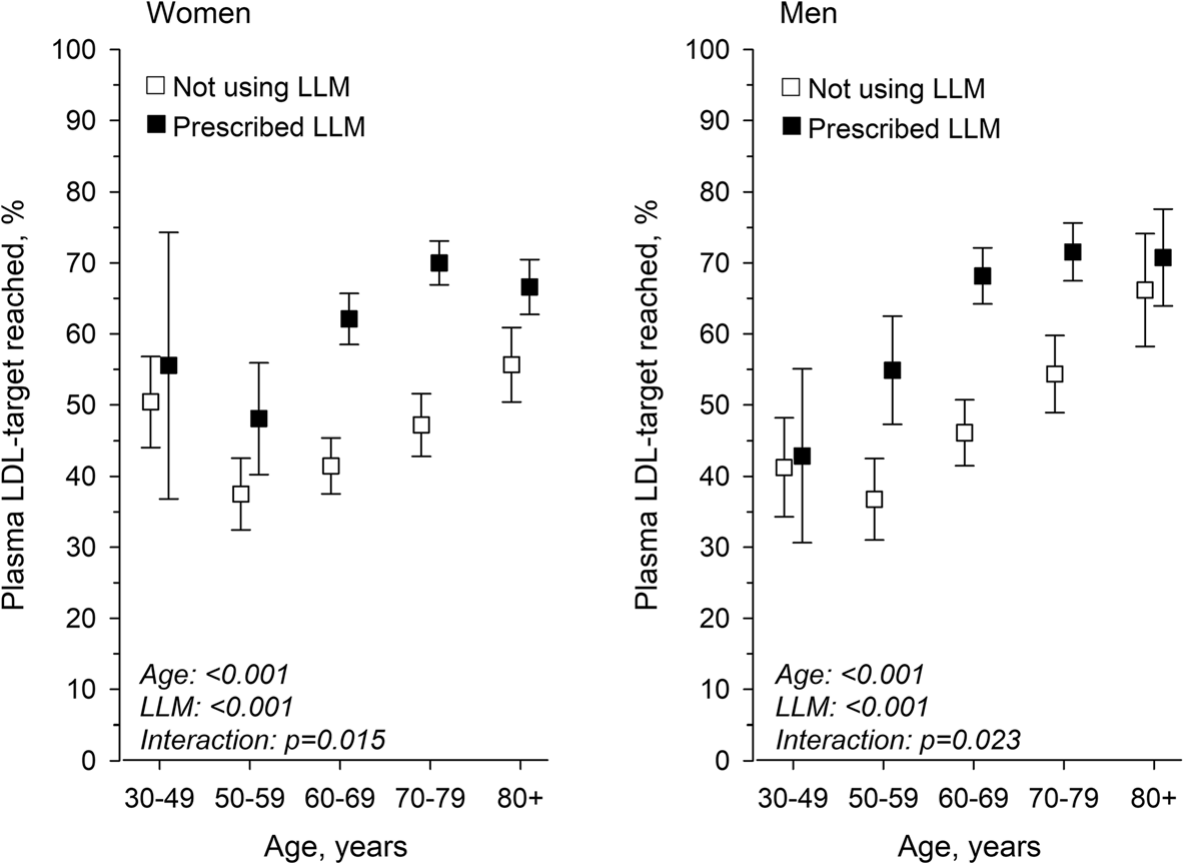
Association between age and proportion reaching LDL-C target. LDL-C: Low-density lipoprotein; LLM: Lipid-lowering medication

The proportion of women and men reaching the LDL-cholesterol target level rose statistically linearly with increasing age (*p*-value for linearity < 0.001). The proportion of patients receiving LDL-C target was higher with the patients with LLM, with the exception of two subgroups: women and men aged 30–49 years, and among men at least 80 years of age (Fig. 2).

**Fig. 3:**
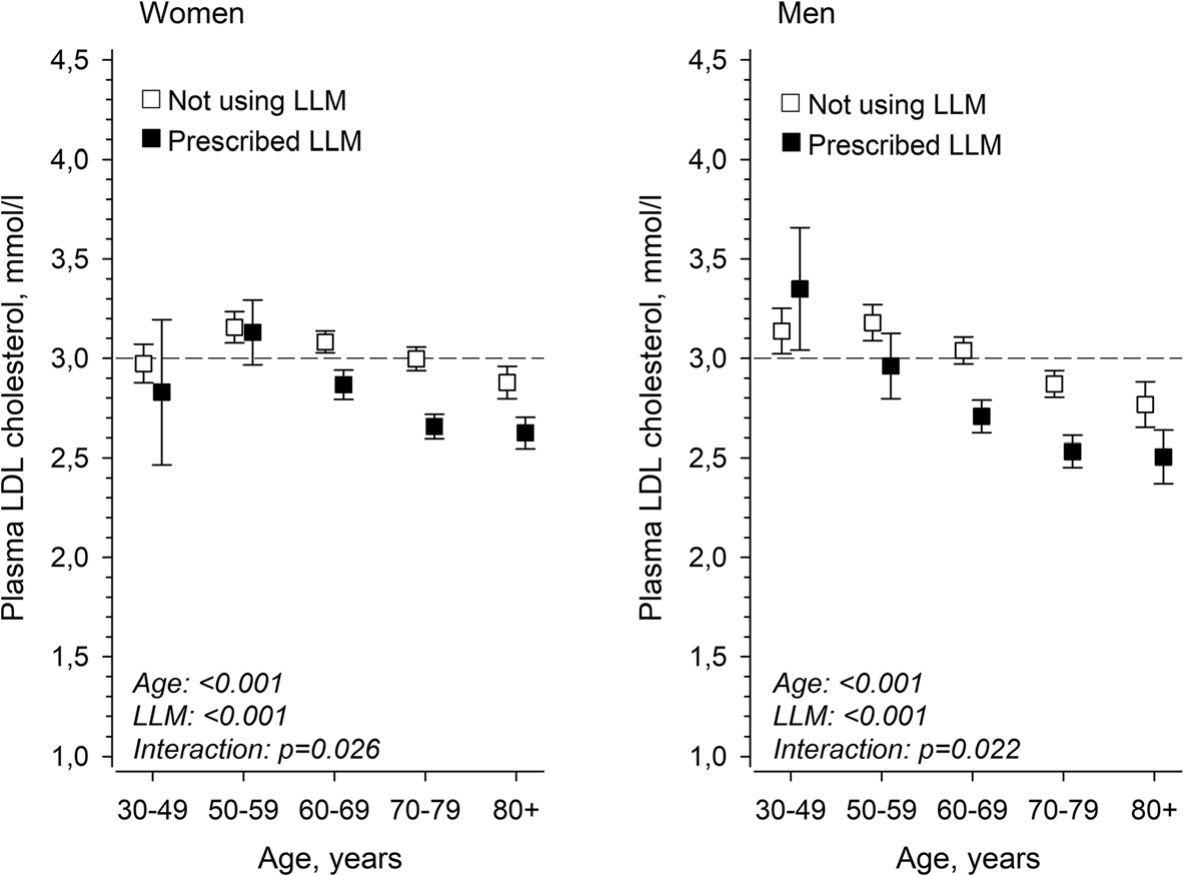
Association between age and plasma LDL-C levels. LDL-C: Low-density lipoprotein; LLM: Lipid-lowering medication

**Fig. 4:**
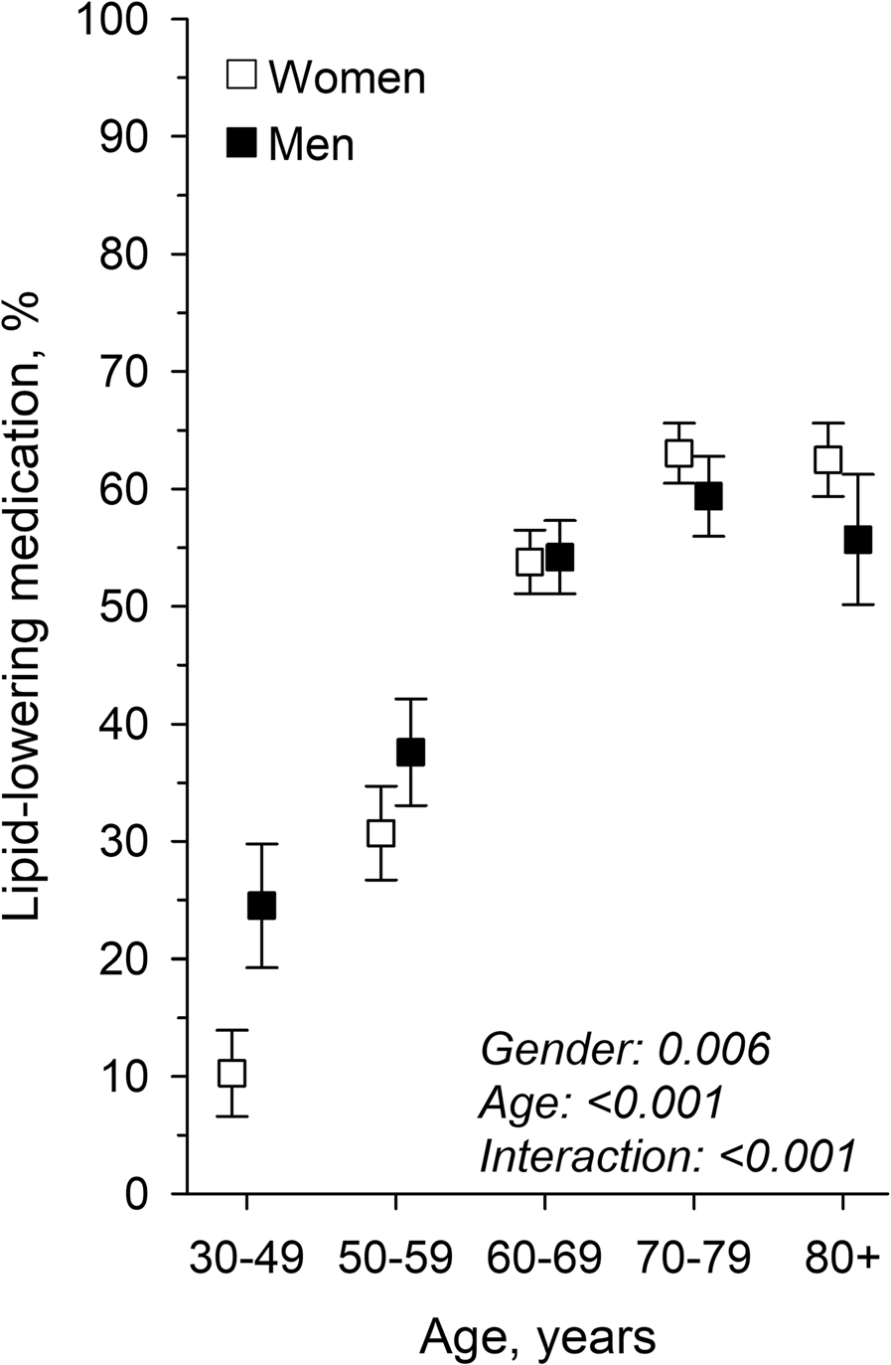
Association between age and lipid-lowering medication use. LDL-C: Low-density lipoprotein; LLM: Lipid-lowering medication

Accordingly, the mean plasma LDL-cholesterol level decreased linearly with increasing age whether LLM was prescribed or not (*p*-value for linearity < 0.001) (Fig. 3). In the age group of 30–49 years, LLM was prescribed to 10.3% of the women and 24.5% of the men. The percentage of patients with LLM rose linearly across older age groups being 63.1% in women and 59.4% in men aged 70–79 years (p-value for linearity < 0.001) (Fig. 4).

## Discussion

Our study indicates that LDL-C control among Finnish hypertensive patients is insufficient, especially among younger patients. Without LLM, more than half of patients did not reach LDL-C target and even with medication, one third of patients did not meet the target. Furthermore, the proportion of individuals reaching LDL-C target seems to be lowest among working age patients who might benefit the most from CVD risk reduction over time [17, 18].

It is clear that younger patients have significantly lower total CVD risk than older patients when assessed using conventional short-term (generally 10-year) risk estimates. Due to current emphasis on short-term risk estimates, clinicians often choose not to initiate effective dyslipidemia treatment when short-term risk is low due to young age. It is remarkable, however, that all our study patients had at least one major CVD risk factor (treatment for hypertension), indicating that proper treatment of another major risk factor (hypercholesterolemia) would decrease the lifetime risk of CVD considerably [18].

Furthermore, it is challenging to rationalize why patients who are on LLM treatment are not treated to a relatively easy-to-reach LDL-C target of < 3 mmol/l, regardless of age. With these individuals, the question is not “Should we treat cholesterol with drugs or not?” but rather: “Should we use the chosen medication properly or not?”. Poor medication adherence often forms a barrier for successful therapy, together with clinical inertia [3, 24, 25]. We argue, however, that lack of sufficient, individual physician feed-back and robust leadership engagement to overcome clinical inertia are also major, but modifiable reasons for this failure. Computerized decision support systems could offer one way to drive change for the better, but feedback alone is not sufficient for system-wide change [26, 27].

### Strengths and limitations

This study has several strengths. To our knowledge, this is the first article to focus on age dependence in LDL-C control among hypertensive patients. Furthermore, Finland has robust public health care and majority of hypertensive patients are treated in public primary health care [28]. To conduct the study, we were able to rely on comprehensive public health care health records of a total population of over 155,000 individuals living in Central Finland (http://pxnet2.stat.fi/PXWeb/pxweb/en/StatFin/StatFin__vrm__vaerak/010_vaerak_tau_123.px/?rxid=ada87756-a322-4f53-b48e-78fdc85edfa2). Hence, the EHR database used in our study includes the majority of all hypertensive patients treated in this area.

Our study has also some limitations that are worth discussion. This was an observational cross-sectional study using routinely collected health care data together with laboratory data. These data sources have naturally several limitations. First, they do not provide sufficient information to assess total individual CVD risk. Therefore, we focused only in hypertensive population without CVD, diabetes or severe renal dysfunction. It is therefore reasonable to assume that LDL-C treatment targets of the general population are used in these patients [3, 9, 23]. Second, these data sources lack trustworthy information on smoking and current blood pressure status. This would be a major problem in prognostic study setting, but is not essential when studying the treatment status of an independent risk factor, such as LDL-C. Smoking and the blood pressure level of hypertensive patients do not change the LDL-C target levels, either. Third, the coverage and accuracy of diagnostic codes is never perfect, and may have resulted in misclassification of some patients, especially diabetics. However, the EHR data used in the study has also been a basis of a rigorous quality measurement system for several years and the accuracy of diagnostic codes has therefore enhanced remarkably. When analysing the data, we were also able to perform multiple chart reviews and found no signs of major misclassification. It is also possible that better target achievement in older age groups is partly due to more frequent use of health services due to increased multimorbidity, but we were not able to detect and compare our findings with the use of health services. Furthermore, it is probable, that some working age patients visit both primary and occupational health care and their LDL-C level may thus be treated to target in occupational health care after being first measured in primary health care. Finally, the status of LLM treatment in this study was based on up-to-date information of LLM prescription but our data does not provide reliable information on the adherence to medication or non-medical treatment of dyslipidemia.

### Comparison with existing literature

Some previous studies have observed a similar age dependent trend in the proportion of individuals reaching LDL-C target [11–14]. It might seem reasonable to assume that physiological changes of aging could explain the better LDL-C control among older patients. However, it has been shown that both plasma LDL-C and total cholesterol levels increase progressively after age of 20 years [29, 30]. One explanation seems to be better LLM adherence among older patients [31]. However, the pattern of age-dependent increase in LDL-C is different between men and women and may thus, at least partly, explain the somewhat weaker association of age and LDL-C -levels among women [32].

Earlier, the gender differences in the proportion of men and women reaching LDL-C target have already raised a need to pay special attention to treatment of dyslipidemia in women [14]. Our results suggest that it is now time to pay more attention to younger dyslipidemia patients, as well. This is further emphasized by earlier research findings indicating that younger age is also associated with lower awareness and treatment rates of elevated blood pressure in Finland [33]. Lifetime risk estimates of CVD are developing rapidly and open access lifetime risk calculators are already available [17, 34]. At least one on-going randomised, controlled clinical study is currently investigating 10-year benefits of statin treatment in 35–59 year-old patients with LDL-C > 1.8 mmol/L and at least one risk factor other than dyslipidemia [16].

## Conclusions

Our findings suggest that working age patients might be the most potential subgroup to enhance LDL-C control and CVD risk reduction among Finnish hypertensive primary health are patients. Focusing more on the lifetime risk of CVD might help primary care physicians to make better informed decisions on dyslipidemia treating among younger hypertensive patients. This should, however, be further examined in different patient populations.

## Data Availability

The datasets generated and analysed during the current study are not publicly available due to protection of individual privacy, but are available from the corresponding author on reasonable request.

## Abbreviations

CVD: Cardiovascular diseases
CKD: Chronic kidney disease
EAS: European Atherosclerosis Society
eGFR: Estimated glomerulus filtration rate (CKD-EPI equation)
EHR: Electronic health record
ESC: European Society of Cardiology
LDL-C: Low-density lipoprotein
LLM: Lipid-lowering medication
